# *MiR-125b* orchestrates cell proliferation, differentiation and migration in neural stem/progenitor cells by targeting *Nestin*

**DOI:** 10.1186/1471-2202-13-116

**Published:** 2012-09-28

**Authors:** Yi Cui, Zhifeng Xiao, Jin Han, Jie Sun, Wenyong Ding, Yannan Zhao, Bing Chen, Xiaoran Li, Jianwu Dai

**Affiliations:** 1 Key Laboratory of Molecular Developmental Biology, Institute of Genetics and Developmental Biology, Chinese Academy of Sciences, Beijing, 100080, China; 2 Reproductive and Genetic Center of National Research Institute for Family Planning, Beijing, 100080, China; 3 Division of Nanobiomedicine, Suzhou Institute of Nano-Tech and Nano-Bionics, Chinese Academy of Sciences, Suzhou, 215123, China; 4 Department of Biochemistry, Dalian Medical University, Dalian, 116044, China

**Keywords:** Neural stem/progenitor cells, *MiR-125b*, *Nestin*

## Abstract

**Background:**

The emerging concept is that microRNAs (miRNAs) play a central role in controlling stem cell self-renewal and fate determination by regulating the expression of stem cell regulators. *miR-125b*, one of neuronal miRNAs, recently was found to be necessary for neural differentiation of neural stem/progenitor cells (NS/PCs). However, the other specific biological role of *miR-125b* in NS/PCs is little known. We used rat NS/PCs as a model system to study the role of *miR-125b* in governing the behavior of NS/PCs.

**Results:**

We report here the transfection of exogenous *miR-125b* inhibited proliferation of NS/PCs but promoted differentiation and migration. Whereas anti-miR-125b had the opposite effect. Similar results were observed when *Nestin* was knocked down by siRNA. Subsequently, we demonstrated that *Nestin* was a direct functional target of *miR-125b*. *MiR-125b* downregulates the expression of luciferase through *Nestin* 3’untranslated region (3’-UTR), and the regulation was abolished by mutations in the *miR-125b* binding site. *MiR-125b* targeted the 3'-UTR of *Nestin* and reduced the abundance of *Nestin* at both mRNA and protein levels.

**Conclusion:**

The results provided new insight into the function by which *miR-125b* modulates NS/PCs proliferation, differentiation and migration. The data also indicated the regulatory role of *miR-125b* in NS/PCs might through the suppression of *Nestin* expression.

## Background

Neural stem/progenitor cells (NS/PCs) are the self-renewing, multipotent cells that generate the main phenotypes of the nervous system [1]. Neural stem/progenitor cells (NS/PCs) have been studied extensively with the hope of using them clinically. Recent studies have shown that NS/PCs can act as promising and beneficial adjuncts for therapy of neurological disorders including brain trauma or spinal cord injuries, etc. Although the functional properties of NS/PCs have been studied extensively, the molecular mechanisms underlying neural stem cell self-renewal, differentiation and migration are not fully understood. To better understand the complex biological regulatory mechanisms of NS/PC will facilitate their clinical application in future.

The discovery of miRNAs opens new possibilities in terms of modulation of stem cells lineage commitment and differentiation [2-4]. As a recently recognized class of small noncoding RNA molecules, miRNAs are epigenetic modulators of mRNA degradation or protein translation. Functional studies indicate that miRNAs participate in the regulation of a number of cellular processes, including development, proliferation, and differentiation [5,6]. Modulating miRNA expression offers new pathways for post-transcriptional gene regulation and stem cell commitment. Several miRNAs have been implicated in regulating self-renewal of neural stem cells and neuronal fate specification [7]. Many studies demonstrated that in vitro transient overexpression or inhibition of brain-specific miRNAs in stem cells significantly directed differentiation along neuronal cell lineages [8]. Unlike messenger RNAs (mRNAs), miRNAs do not encode proteins, but rather bind mRNAs, regulating their stability and translation into proteins. A single miRNA can target hundreds of mRNAs resulting in very large effects on the molecular constitution of cells. Thus, it is vital to understand the unique functions and targets of miRNA.

*MiR-125b*, a brain-enriched miRNA, is highly expressed in the central nervous system (CNS), including the brain and spinal cord. As a homolog of lin-4 (82% identical), *miR-125b* is highly conserved from flies to humans (100% identical) [9]. Upon neural differentiation, *miR-125* as a key player in the molecular cascade that contributes to the irreversible commitment of pluripotent human stem cells to the neural lineage [10]. Minh T et al. have also demonstrated that the ectopic expression of miR-125b increases the percentage of differentiated SH-SY5Y cells with neurite outgrowth. Their report demonstrates that *miR-125b* is important in regulating neuronal differentiation [11]. In addition, it has also been demonstrated that *miR-125b* was up-regulated during neural differentiation of embryonic stem (ES) cells and embryo carcinoma (EC) cells. *MiR-125* was identified as being involved in mechanisms governing the repression of self-renewal in embryonic stem cells [12]. Taken together, these observations suggest that *miR-125b* may act as an ancient regulatory switch for neuronal differentiation in stem cells.

In the present study, we systematically investigated the functions of miR-125b in NS/PCs and found new downstream target of miR-125b. We demonstrated the important role of *miR-125b* in coordinating the proliferation and differentiation of NS/PCs using both knockdown and ectopic expression approaches. We also identified *miR-125b* promotes migration during the early stages of neuronal differentiation. Furthermore, our studies first demonstrated that *miR-125b* suppresses the expression of *Nestin* by binding to the 3'-UTR of its mRNAs. As a widely employed marker of multipotent neural stem cells, *Nestin* was involved in a number of cellular processes, such as proliferation, differentiation, migration [13,14]. Our study presented here revealed a previously undescribed link between *miR-125b* and an essential stem cell regulator *Nestin*, identified it as a key target of *miR-125b* in NS/PCs.

## Results

### *MiR-125b* expression increases during neural stem cell differentiation

**Figure 1:**
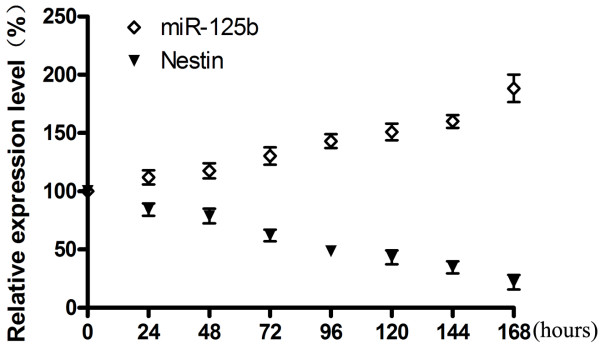
**Expression pattern of *miR-125b *and *Nestin *in NS/PCs during differentiation.** Expression pattern of *miR-125b* in NS/PCs during differentiation quantified by real-time PCR using specific Taqman primers. *U6* was used as the internal control. In the meantime, the expression level of *Nestin* was quantified by real-time PCR during NS/PCs differentiation. *GAPDH* was used as the internal control. Data represent mean±SD of three different experiments.

NS/PCs may give rise to neural and neuronal progenitor cells, it has been demonstrated that the dynamic changes in the expression of miRNA occurs during neuronal differentiation. In order to understand the role of *miR-125b* in the regulation of neuronal differentiation, the expression pattern of *miR-125b* was performed by real-time PCR. The results showed that the expression pattern of *miR-125b* was up-regulated in a time-dependent manner with neural differentiation proceeded suggesting that *miR-125b* may be involved in differentiation of NS/PCs (Figure 1). Our results are coincident with previous reports indicating that *miR-125b* was up-regulated during neural differentiation process [10-12].

### *MiR-125b* Inhibits *Nestin* Expression by Binding to its 3’-UTR

**Figure 2:**
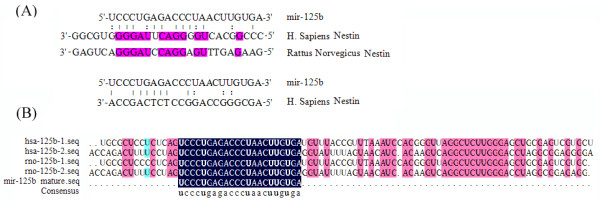
**Identification of a *miR-125b *target site in the 3’UTR of *Nestin *mRNA.** (**A**) Putative binding sites of *miR-125b* in the *Nestin* 3’UTR predicted by Microcosm Targets in different species. The nucleotide pairing of *miR-125b* and *Nestin* 3'-UTR. The vertical lines indicate perfect pairing, while ":" indicates G-U wobble. (**B**) Conservation of *miR-125b* gene sequences between human and rat. Sequence alignment indicates that *miR-125b* is 100% conserved in human and rat.

Toward understanding the role of *miR-125b* involved in regulating wide array of cellular process in NS/PCs, it is therefore important to identify its target genes. Using the MicroCosm Targets (formerly miRBase Targets), *miR-125b* was identified to have a conserved target site in the 3’-UTR of *Nestin* gene (Figure 2A). This program predicted a putative binding site for *miR-125b* in the *Nestin* 3’-UTR. *MiR-125b* is highly conserved in vertebrates, and its mature nucleotide sequence is 100% identical among several species (Figure 2B). Although 3’-UTR sequences of *Nestin* are not highly conserved in mammals, the putative binding sites for *miR-125b* in *Nestin* are also found in both human and rat (Figure 2B).

**Figure 3:**
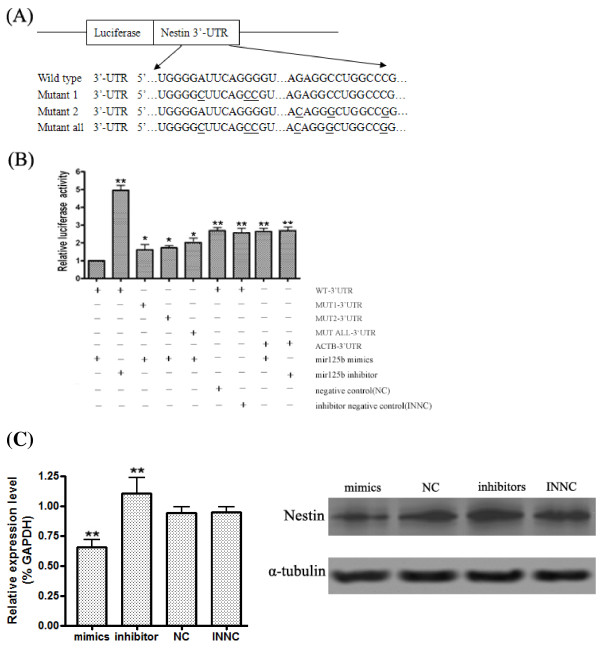
***MiR-125b *suppresses the expression of the luciferase reporter with *Nestin *3’-UTR.** (**A**) Schematic representation of the constructs used in the luciferase reporter assay. The *Nestin* 3’-UTR sequences containing the putative binding sites of *miR-125b* were inserted into the the pGL3 luciferase reporter vector. Site-directed mutagenesis was performed to mutate base pairs in the predicted seed region targeted by *miR-125b* in the *Nestin* 3'-UTR. The mutant binding site is underlined. (**B**) Luciferase assay was performed 72 hours after transfection using the Dual Luciferase. *MiR-125b* down-regulated luciferase activities controlled by wild-type *Nestin* 3’UTR, but did not affect luciferase activity controlled by mutant *Nestin* 3’UTR. The firefly luciferase activity was standardized to renilla luciferase control. (**P < 0.01 vs. WT; *P < 0.05 vs. WT).The results are means of three independent experiments±SD. (**C**) The expression level of *Nestin* was analysed by qRT-PCR and the Western blot in NS/PCs after transfection with miR-125b mimics, inhibitors, NC and INNC. Bars show mean ± SD. All experiments were repeated three times. * P < 0.05 vs. ctr, ** P < 0.01 vs. ctr.

To further validated the interaction between *miR-125b* and the *Nestin* 3’-UTR, the luciferase reporter system was used. We constructed vectors containing wild-type or mutant 3’-UTR of *Nestin* directly fused to the downstream of the Firefly luciferase gene to verify that the putative *miR-125b* binding site in the 3’-UTR of *Nestin* is responsible for regulation by *miR-125b* (Figure 3A). The wild-type or mutant vector of *Nestin* was cotransfected into Hela cells with *miR-125b* mimics, *miR-125b* inhibitors, *miR-125b* NC (negative control), *miR-125b* INNC (inhibitor negative control). All of them are 2'-O-methyl (2'-O-Me) modified RNA oligonucleotides chemically synthesized by Shanghai GenePharma Co.,Ltd, 2'-O-Me modification is frequently used to protect oligoribonucleotides from degradation in cultured cells. The transfection efficiency was normalized by co-transfection with Renilla reporter vector. When *miR-125b* mimics were co-transfected with the *Nestin* reporter constructs, luciferase activity was significantly suppressed compared with cotransfection with *miR-125b* inhibitors, NC and INNC. However, *miR-125b* mimics were unable to suppress luciferase expression with the mutant *Nestin* 3’-UTR which the putative binding sites of *miR-125b* were mutated (Figure 3B). Considering that there still lies some complementary sequence to miR-125b in the *Nestin* 3'-UTR besides the bases we have mutated, the binding force between miR-125b and the Nestin 3'-UTR was weakened but not eliminated. Thus the luciferase expression in the mutated vector group was a little suppressed compared to NC and INNC group. Furthermore, we constructed the 3'-UTR vectors of ACTB and then cotransfected the vector into Hela cells with miR-125b mimics and inhibitors. The results indicated that miR-125b mimics and miR-125b inhibitors had little effect on luciferase expression with the ACTB 3’-UTR. These experiments demonstrated that *miR-125b* interacts directly with the binding site in *Nestin* 3’-UTR to regulate luciferase reporter expression. Taken together, these findings indicated that *Nestin* is a direct downstream target for *miR-125b* in NS/PCs.

Subsequently, we transfected *miR-125b* mimics, *miR-125b* inhibitor, NC and INNC in NS/PCs to evaluate whether *Nestin* mRNA and protein levels are directly affected by the activation of *miR-125b*. Three days post transfection, the *Nestin* mRNA and protein level were analyzed by real-time PCR and Western blot (Figure 3C). Transfection with *miR-125b* mimics led to down-regulation of *Nestin* mRNA and protein in NS/PC cells compared with NC, whereas treatment with *miR-125b* inhibitor induced the expression of *Nestin* increase in NS/PC cells compared with INNC. Taken together, these experiments support the hypothesis that *Nestin* is a direct target of *miR-125b* and that *miR-125b* activation in NS/PCs elicits a decrease of *Nestin* protein.

### *MiR-125b* inhibits NS/PCs proliferation

**Figure 4:**
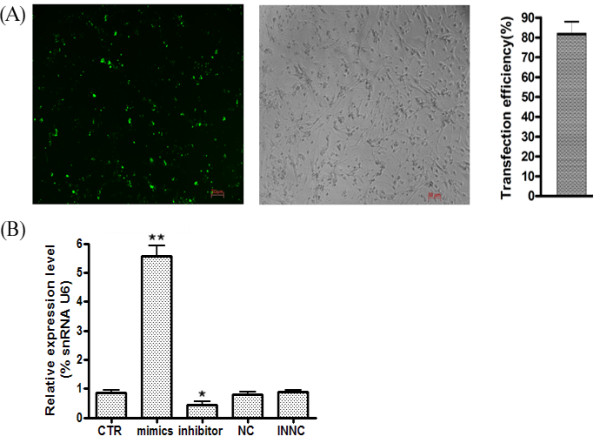
**Efficient transfection of *miR-125b *in NS/PCs.** (**A**) At 24 post-transfection, transfection efficiency was assayed via fluorescent detection of the 5’-FAM labelled *miR-125b* mimics in living NS/PCs. The high transfection efficiency was demonstrated using fluorescent microscopy. (**B**) Real-time PCR was used to assess the efficiency of *miR-125b* transfection at 72 h after differential transfecton. *U6* was included as a loading control.

**Figure 5:**
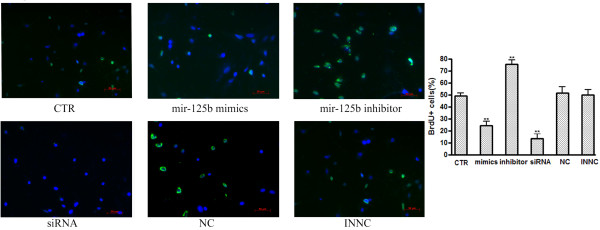
***miR-125b *inhibit NS/PCs proliferation.** Cell proliferation was evaluated by bromodeoxy uridine (BrdU) incorporation. Representative images of NS/PCs stained with Brdu (green) and Hoechst dye (blue) 72 h after transfection. After transfection, the NS/PCs were maintained in growth medium until they were fixed and stained. Images were acquired by a microscope using a 20 × objective lens. The percentage of BrdU-positive cells at 72 h after transfection is shown. Bars show mean ± SD. All experiments were repeated three times. * P < 0.05 vs. ctr, ** P < 0.01 vs. ctr.

In an attempt to investigate whether *miR-125b* influences the proliferation of NS/PCs, we performed BrdU labeling on rat NS/PCs after transfection of *miR-125b* mimics, *miR-125b* inhibitor, NC and INNC. Considering the tightly packed cells of a neurosphere are difficult to transfect, the neurospheres were mildly trypsinized into single cells and then seeded in polyornithine-laminin coated 24-well plate. When cells reached 70% confluence, the *miR-125b* mimics, *miR-125b* inhibitor, NC and INNC was transfected into the cells. The transfection efficiency was observed under the fluorescent microscope. Transfection of NS/PCs with a FAM-labeled *miR-125b* mimics indicated the high efficiency of transfection after 24 hours (Figure 4A). We also evaluated transfection efficiency by detecting the relative expression of *miR-125b*. As expected, the intracellular level of *miR-125b* was elevated by transfection of *miR-125b* mimics, and was decreased by the transfection of *miR-125b* inhibitors, showing that the cellular level of *miR-125b* could be controlled by the transfection of *miR-125b* mimics and *miR-125b* inhibitors (Figure 4B). Therefore we used BrdU labeling to investigate the role of *miR-125b* in cell proliferation, BrdU was added 16 hours prior to stem cell harvesting and then stained with an anti-BrdU antibody. Immunostainings for BrdU show that the up-regulation of miR-125b inhibited the proliferation of NS/PCs (Figure 5).

### *MiR-125b* accelerates neural differentiation of NS/PCs

**Figure 6:**
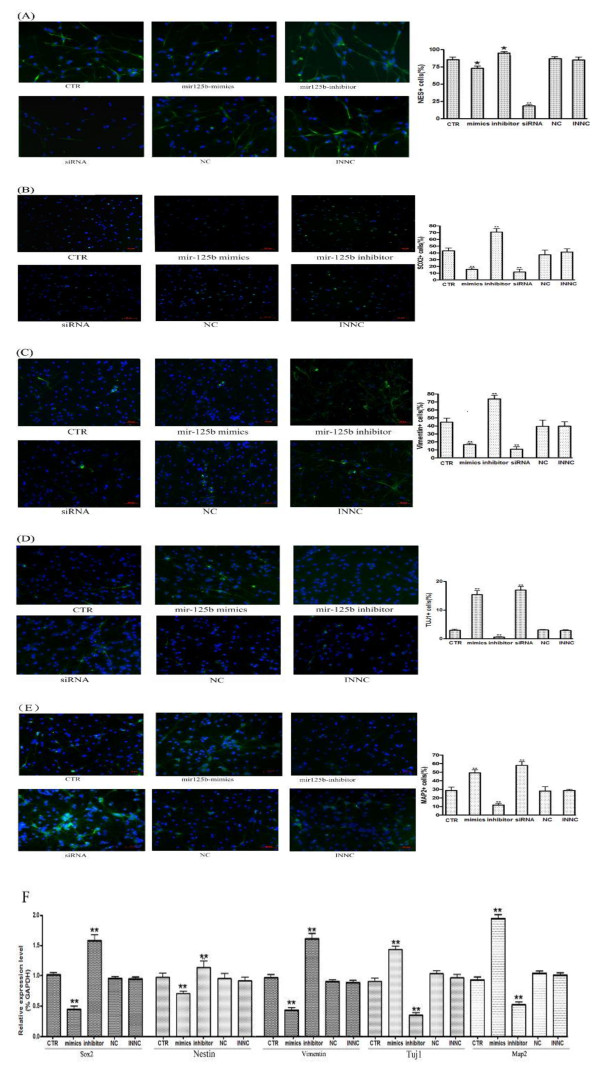
***miR-125b *accelerates neural differentiation.** After transfection, the NS/PCs were maintained in differentiation medium. The expression of the NS/PCs progenitor marker *Nestin *(**A**), *Sox2 *(**B**), *Vinemetin* (**C**) and the neuronal marker *Tuj1 *(**D**), *Map2 *(**E**) were detected by immunofluorescence. Images were acquired by a microscope using a 20 × objective lens. Bars show mean ± SD. All experiments were repeated three times. * P < 0.05 vs. ctr, ** P < 0.01 vs. ctr. (**F**) Real-time PCR analysis of *Nestin*, *Sox2*, *Vinemetin*, *Tuj1* and *Nestin* expression in the same differentiation time course 72 h after transfection. *GAPDH* was included as a loading control. Bars show mean ± SD. All experiments were repeated three times. * P < 0.05 vs. ctr.

We next examined whether *miR-125b* influences the differentiation of NS/PCs. The role of *miR-125b* in promoting the differentiation of NS/PCs was demonstrated further by staining for neuronal markers. Following transfected with *miR-125b* mimics, *miR-125b* inhibitors, NC, INNC, NS/PCs were cultured in differentiation conditions for 72 h. A significant decrease in the number of *Nestin*-positive cells, *Sox2*-positive cells and *Vimentin*-positive cells was found in NS/PCs transfected with *miR-125b* mimics compared to control group (Figure 6A, B, C). On the other hand, The percentage of *Tuj1*-positive cells and *Map2*-positive cells in NS/PCs transfected with *miR-125b* mimics were significantly higher than control group (Figure 6D, E). The results indicated that *miR-125b* over-expression enhances rate of neuron differentiation, by contrast, knockdown of *miR-125b* decreases rate of neuron differentiation compared to the control. The results of real-time PCR assays were well consistent with the results of immunofluorescence indicating that overexpression of *miR-125b* can independently increase the expression of neuronal marker *Tuj1* and *Map-2* but decrease the expression of *Nestin*, *Sox2* and *Vimentin* (Figure 6F).

### *MiR-125b* promotes the migration of NS/PCs

**Figure 7:**
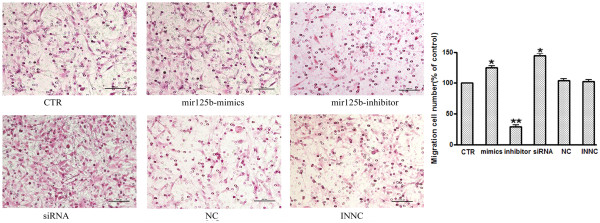
***MiR-125b *enhanced the mobility ability of NS/PCs cells using transwell migration assay.** Migration assays were performed after 72h transfection. Quantitation and representative photomicrographs showing that *miR-125b* promote cell migration in NS/PCs. Bars show mean±SD. All experiments were repeated three times. * P < 0.05 vs. ctr.

We used transwell migration assay to compare the in vitro migratory capacities of NS/PCs among groups transfected with *miR-125b* mimics, *miR-125b* inhibitor, NC, INNC. Migration assays were performed after 72h transfection. The percentage of migrated cells was normalized to that of control cells. The results indicated that overexpression of *miR-125b* led to a increase in cell migration whereas knockdown of *miR-125b* led to a reduction in cell migration compared to control group in NS/PCs (Figure 7).

### Inactivation of *Nestin* not only reduces cell proliferation but promotes neural differentiation and migration in NS/PCs

Consistent with previous reports,we detected the relative expression of *Nestin* mRNA decreased during neuronal differentiation of NS/PCs in our studies (Figure 1). We considered the possibility of these biological changes induced by *miR-125b* may be due to the down-regulation of *Nestin*. For this purpose, we knocked down *Nestin* by RNAi and assayed the effect of *Nestin* knockdown on cell proliferation, differentiation and migration in NS/PCs (Figures 5, 6, 7). The results indicated that knockdown of *Nestin* reduced cell proliferation and promoted neural differentiation and migration in NS/PCs. The siRNA transfected NS/PCs showed a similar biological behavior as observed in the cells transfected with *miR-125b* mimics. Taken together, our data suggested that *miR-125b* regulates neural stem cell proliferation, differentiation and migration through targeting *Nestin* expression.

### MiR-125b regulate NS/PCs activities by targeting Nestin

**Figure 8:**
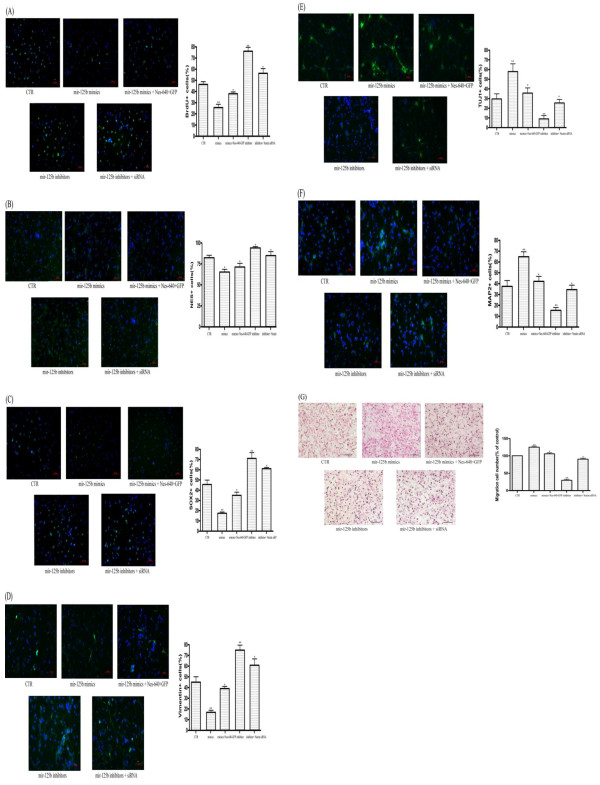
***MiR-125b *regulates NS/PCs activities by targeting *Nestin.*** (**A**) The Brdu incorpation assay indicated that overexpress Nestin may rescue the inhibitory effect on proliferation which *miR-125b* mimics bring about. Also, the *Nestin* siRNA may decrease the stimulus effect on proliferation which *miR-125b* inhibitors bring about. (**B-F**) The immunofulorescence results indicated that overexpress Nestin may rescue the stimulus effect on NS/PCs differentiation which *miR-125b* mimics bring about. Also, the *Nestin* siRNA may weaken the inhibitory effect on NS/PCs differentiation which *MiR-125b* inhibitors bring about. (**B**) *Nestin*, (**C**) *Sox2*, (**D**) *Vimentin*, (**E**) *Map2*, (**F**) *Tuj1.* (**G**) The migration assay indicated that overexpress *Nestin* may rescue the stimulus effect on NS/PCs migration which *miR-125b* mimics bring about. Also, the *Nestin* siRNA may weaken the inhibitory effect on NS/PCs migration which *miR-125b* inhibitors bring about. Bars show mean ± SD. All experiments were repeated three times. * P < 0.05 vs. ctr, ** P < 0.01 vs. ctr.

In order to further validate the function of miR-125b in NS/PCs activities via direct regulating Nestin, we transfected NS/PCs with miR-125b mimics and the Nestin overexpressed plasmid (Nes-640-GFP) simultaneously. The plasmid Nes-640-GFP was demonstrated to have a longer half-life and more prominent protein levels in cells, which was kindly provided by John E. Erikssona (Turku Center for Biotechnology, University of Turku and Åbo Akademi University, Turku, Finland) (Sahlgren et al., 2006). We demonstrated that exogenously expressed Nestin could rescue the effect on NS/PCs proliferation, differentiation and migration resulting by miR-125b mimics (Figure 8). Furthermore, we determined to test whether depletion of Nestin may weakened the role of miR-125b inhibitors in NS/PCs. When Nestin was knocked down by siRNA, the stimulus effect on cell proliferation and the inhibitory effect on differentiation and migration which miR-125b inhibitors bring about was weakened (Figure 8). Hence, our data support the idea that Nestin as a downstream target of miR-125b was implicated in NS/PCs activities, such as proliferation, differentiation and migration.

## Discussion

It is well known that NS/PCs contribute to the neurogenesis of the central nervous system. During central nervous system development in vertebrates, neural stem cell fate are strictly controlled under regional and temporal manners [15]. Besides the discovery of NS/PCs in the embryo, recent studies have substantially demonstrated that NS/PCs continue to reside in the adult nervous system such as hippocampus, the subventricular zone (SVZ), and in some non-neurogenic regions, including spinal cord [1,16]. NS/PCs persist throughout adult life in nervous system in order to replace the mature cells that are lost to turnover, injury, or disease. In response to CNS injuries, NS/PCs can migrate to areas of injury and differentiate into neuronal and glial cell types, thus are likely to contribute to the repair of damaged tissue. Therefore, unraveling the mechanisms of cellular processes in NS/PCs will provide insights into both basic neurosciences and clinical applications of stem cell-based cell replacement therapies.

Although it is well established that NS/PCs have the potential to cure many diseases, the molecular basis of this process, particularly the role of miRNAs, remains largely unknown. MiRNAs are a class of small RNA regulators that are involved in numerous cellular processes, including development, proliferation, differentiation, and plasticity. Emerging evidence has shown that the process of neural stem cell self-renewal, fate specification and migration can be regulated by miRNA [17]. As mentioned above, there is growing evidence to suggest a role for *miR-125b* during neuronal differentiation. In our study we first quantified the relative expression levels of *miR-125b* during neural differentiation. It is likely that the distinct temporal expression patterns of *miR-125b* reflect its specific roles in coordinating gene expression profiles that characterize neural cell fate determination. Real-time PCR analyses indicated that *miR-125b* is significantly up-regulated during the differentiation of NS/PCs. Our study presented here revealed that the inhibition of *Nestin* expression by miR-125b provides double insurance to inhibit NS/PCs proliferation when miR-125b expression is elevated upon differentiation. Moreover, our results suggested that *miR-125b* promote migration during the early differentiation of NS/PCs.

The functions of animal miRNAs may be elucidated through their targeted genes. To gain a better understanding the role of *miR-125b* involved in regulating wide array of cellular processes in NS/PCs, it is necessary to identify its target genes. A single miRNA can recognize hundreds of targets. However, one or two of these target genes may be more important targets in specific cellular context [18]. Data from computational analyses suggest that *Nestin* may be regulated by *miR-125b*. As a widely employed marker of multipotent NS/PCs, *Nestin* is important for the proper survival and self-renewal of NS/PCs. Numerous in vivo and in vitro studies now rely on *Nestin* expression to track the proliferation, migration and differentiation of NS/PCs [13]. Computer analysis predicted that *miR-125b* had partial sequence complementarity within the 3’-UTR region of the *Nestin* mRNA. Using a luciferase assay, we showed that *miR-125b* directly target *Nestin* through binding a specific site in its 3’-UTR in NS/PCs. Since miRNAs have been shown to regulate gene expression through both translational attenuation and mRNA degradation. Both real-time PCR and western blot data suggests that *miR-125b* could repress *Nestin* expression at both the mRNA and protein levels via directly binding to its 3'-UTR. In line with previous report, we detected the expression of *Nestin* is high in NS/PCs but it is reduced dramatically upon differentiation. In contrast, the level of *miR-125b* is increased significantly upon differentiation. In this case, *Nestin* is preferentially expressed at high levels when the targeting miRNA expression is low. The expression of this target is down-regulated as *miR-125b* accumulates. Taken together, we identified *Nestin* as a target of *miR-125b* in NS/PCs by using a bioinformatics approach and experimental validation.

It has previously been reported that *miR-125b* is a positive regulator of differentiation of human neural progenitor ReNcell VM (RVM) cells [19], but the underlying mechanism of how it regulates proliferation is not known. Accumulating evidence has indicated that *Nestin* may actively functions in cell growth or proliferation of different types of neural stem cells. The specific interfering role of N*estin* siRNAs was in vitro identified on cell growth of C6 astrocytoma cells, suggesting that siRNA-induced suppression of central neural system tumor cell growth [19]. To support the hypothesis that the effect of *miR-125b* on cellular process is mediated largely through *Nestin*, we also knocked down *Nestin* expression in NS/PCs using its sequence-spcific siRNA. By using BrdU incorporation assays, we demonstrated that overexpression of *miR-125b* led to dramatically reduced NS/PCs proliferation in line with the results of *Nestin* siRNA-treated cells, indicating that *miR-125b* inhibit NS/PCs proliferation by targeting *Nestin*.

Using immunostaining for the neural marker, we evaluated the effect of *miR-125b* on neural differentiation potential of NS/PCs. Overexpression of *miR-125b* resulted in the percentage of *Tuj1* and *Map-2* positive cells increased, whereas that of *Nestin*-positive cells decreased. There was no difference in the percentage of *GFAP* positive cells among all groups (data not shown). This could be explained by the fact that most NS/PCs were also expressed glial marker *GFAP*. In the meantime, decrease of *Nestin* by RNAi strikingly accelerated the neural differentiation process, suggesting that *miR-125b* promoted differentiation might be mediated by *Nestin*. Also, we provided direct evidence that *miR-125b* is a positive regulator of migration during early neuronal differentiation of NS/PCs. The results are in good agreement with previous findings demonstrating that exogenous *miR-125b* expression increased migration of ishikawa cells and abrogating expression of *miR-125b* suppressed migration of AN3CA cells in vitro [20]. Previous results implied that N*estin* might serve as a scaffold regulating the activity of the *Cdk5/p35* signaling complex [21,22]. *Cdk5* is involved in the processes of neuronal differentiation and migration [23,24]. Given that knockdown of *Nestin* was sufficient to activate *cdk5* anctivity, *miR-125b* promote neural differentiation and migration might via activate *cdk5* activity during early neuronal differentiation of NS/PCs. A recent report by Donghyun Park et al. has shown that the neural stem cells which derived from *Nestin* knockout mice showed dramatically reduced self-renewal ability but no overt defects in cell proliferation or differentiation [13]. We speculated the discrepancy between our study and their study lies in the NS/PCs isolated from the embryo at different development stages and different tissues. In spite of the results of their study were inconsistent with our results, it can be interpreted as the *Nestin* have multiple functions at different developmental stages or in different tissues.

In conclusion, these results support the conclusion that *miR-125b* is implicated in regulating its downstream target genes, *Nestin*, which play a crucial role in NS/PCs proliferation, differentiation and migration. In addition, our study does not exclude the possibility that additional target genes may also play a role in *miR-125b* function in NS/PCs. Cultured NS/PCs hold considerable promise both in terms of their application to a wide variety of research paradigms and in the development of potential therapeutic modalities [25]. Several important breakthroughs during recent years have raised a hope that stem cell-based therapies could be used to restore function and integrity after acute brain injury and other disorders of the central nervous system. A better understanding of the role exerted by *miR-125b* in NS/PCs will contribute to offer promising prospects for their increasing application in the development of new cellular therapies in humans.

## Conclusions

Our data showed the involvement of *miR-125b* in the coordinated regulation of proliferation, differentiation and migration in NS/PCs. The work also indicated that *miR-125b* mediated silencing of *Nestin* may be involved in regulating NS/PCs cell proliferation, differentiation and migration.

## Methods

### Cell culture

Primary neural progenitors were prepared by a modified version of a published protocol [26]. All animal procedures were performed in accordance with Chinese Ministry of Public Health (CMPH) Guide and US National Institute of Health (NIH) Guide for the care and use of laboratory animals and was approved by the IACUC Bioethics Committee (IACUC, Institutional Animal Care and Use Committee-Bioethics Committee) of the Institute of genetics and developmental biology, Chinese academy of sciences. Briefly, neural tissue samples were dissected from the hippocampus of newborn Sprague–Dawley (SD) rat pups, cells were mechanically isolated by repeated trituration in a serum-free culture media composed of Dulbecco's modified Eagle's medium (DMEM) and F12 nutrient (1:1) (Invitrogen, Carlsbad, CA . USA). The dissociated cells were collected by centrifugation at 150 × g for 5 min and were resuspended in DMEM/F12 medium containing bFGF (10 ng/ml), EGF (20 ng/ml), and B27 supplement (Gibco, Invitrogen, Carlsbad, CA. USA). After culturing for 8 days, neurospheres formed and the cells were used for following experiments. The neurospheres were then digested with 0.25% trypsin into single cell suspension and seeded in poly-D-lysine coated 48-well culture plates at a density of 2 × 10^4^ cells per well. NS/PCs was resuspended in adhesion medium containing DMEM, 10% FBS (Gibco, Invitrogen, Carlsbad, CA. USA). For differentiation experiments, the medium was changed to the differentiation medium serum-free DMEM (high glucose) with B27 supplement.

### Construction of reporter plasmids and luciferase assay

The full length 3'-UTR of the human *Nestin* gene and the fragment containing the putative *miR-125b* binding site were amplified from human cDNA and individually cloned into a modified *pGL3*-promoter vector (Promega, Madison, WI, USA). The *pGL3* luciferase reporter vector was modified by adding the restriction sites *SpeI, NaeI, SphI, ApaI* and *Pst*I downstream of the coding region of the firefly luciferase, using PCR amplification with the following primers: forward/*NcoI* 5'-CTGCCATGGAAGACGCCAAAAACATA-3' and reverse/XbaI 5'-TGCTCTAGACTGCAGGGGCCCGCATGCGCCGGCACTAGTTTACACGGCGATCTTTCCGC-3'. The full-length 3’-UTR and the mutated 3’-UTR of *Nestin* was amplified by PCR using the primers listed in Table 1. Also, the 3’-UTR of ACTB was amplified by PCR using the primers listed in Table 1. The mutated 3’-UTR of *Nestin* was generated using site-directed mutagenesis according to the manufacturer's protocol. The primers containing the mutation are listed in Table 1. The site-directed mutagenesis kit (TransGen Biotech, Beijing, China) was used to generate the mutation in the 3' UTR of the *Nestin* gene by PCR, using the full-length 3' UTR of *Nestin* as the template. All PCR products were digested at the *SpeI* and *SphI* sites before cloning into the *pGL3*-promoter vector.

**Table 1:** Primers used to construct luciferase reporter plasmids of Nestin

**Gene name**	**Primer sequence**	**Product size (bp)**
Nestin 3’UTR	Forward/ SpeI: 5'- GGACTAGTGAAAAGACCATCTG -3'	431
(mir-125b sense)	Reverse/ SphI:5'- ACATGCATGCAGGGCGTGCTACTG -3'	
Nestin 3’UTR	Forward/ SpeI: 5’- GCACTGCCGACTTACGGGTGCGGGGAGGGGA -3’	431
(mir-125b mutated 1 putative binding region)	Reverse/ SphI:5'- CACCCGTAAGTCGGCAGTGCCGGGCAGATGG -3'	
Nestin 3’UTR	Forward/ SpeI: 5’- TGGCTGACAGGGCTGGCCGGCTAAGGTGAAA -3’	431
(mir-125b mutated 2 putative binding region)	Reverse/ SphI: 5'- CCGGCCAGCCCTGTCAGCCAGAAACCATATGTCAA -3'	
ACTB 3’UTR	Forward/ SpeI: 5'- GGACTAGTGCGGACTATGACTTAGTTGC-3'	563
	Reverse/ SphI: 5'- ACATGCATGCAAGTCAGTGTACAGGTAAGC-3'	

To assess whether *miR-125b* could bind to *Nestin* mRNA 3' UTR, Hela cells were seeded in 24-well plates in DMEM high-glucose medium. The cells were transfected with 500 ng of wild-type or mutant reporter plasmid, 25 ng of Renilla luciferase expression vector, and *miR-125b* mimic (10nM) or *miR-125b* inhibitor (10nM) or equal amounts of NC, INNC (GenePharma Co., Ltd., Shanghai, China) using Lipofectamine 2000™ reagent (Invitrogen, Carlsbad, CA, USA), according to the manufacturer’s instructions. After 48 h, the cell extract was obtained, and firefly and Renilla luciferase activities were measured with the dual-luciferase reporter system (Promega, Madison, WI, USA) according to the manufacturer's instructions. The results were expressed as relative luciferase activity (Firely LUC/Renilla LUC).

### Transient transfections and cell treatments

To perform RNAi experiment, NS/PCs were transfected with siRNA duplexes when cells reached 80–90% confluency on the day of transfection. Lipofectamine 2000™ reagent was used as the transfection reagent. Three independent transfection experiments were performed in triplicate with each construct. Transfection was carried out after third passage in 6-well plate (Corning Costar, Cambridge, MA). Twenty-four hours before transfection, 3 × 10^5^ NS/PCs per well were seeded in 6-well plate with fresh DMEM/F12 without antibiotics, in order to obtain 80–90% confluency on the day of transfection. For transfection, 10 μM siRNA solution were used for each well according to the siRNA application protocol. The rat N*estin* siRNA contained a pool of three different siRNA duplexes as follows [19]:

(a)GAGAUUUCUGAUUCUCUCUtt (sense)/AGAGAGAAUCAGAAAUCUCtt (antisense)

(b)UGGUCCUCCUCUUCUGGAGtt (sense)/CUCCAGAAGAGGAGGACCAtt (antisense)

(c)GCUGACUGUCCUCUACCCUtt (sense)/AGGGUAGAGGACAGUCAGCtt (antisense)

SiRNA oligonucleotide duplexes were synthesized by Genepharma Biotech (GenePharma Co., Ltd., Shanghai, China). The Lipofectamine siRNA complexes were removed after 6 h and fresh differentiation medium containing antibiotics was added to the cells.

In rescue experiment, NS/PCs were cotransfected with the plasmid NES-640-GFP and *miR-125b* mimics. NS/PCs were transfected with Lipofectamine® LTX with Plus™ Reagent (life technologies, New York, NY) according to the manufacturer’s protocol. For each well of a Lipofectamine LTX transfection, 2.5 μg of NES-640-GFP plamid DNA and 10nM of *miR-125b* mimic were combined with 1.25 μl of Plus reagent (Invitrogen,catalog number 11514-015) in 500 μl of serum-free media for 5 min. 5 μl of Lipofectamine LTX (Invitrogen, catalog number 15338-100) was then added, mixed, and incubated for an additional 25 min before being added dropwise to cells. In another group, NS/PCs were cotransfected with *Nestin* siRNA and *miR-125b* inhibitor, 10 μM *Nestin* siRNA and 10 nM of *miR-125b* mimics were mixed together to perform the cotransfection experiments. Lipofectamine 2000™ reagent (Invitrogen, Carlsbad, CA, USA) was used according to the manufacturer’s instructions as mentioned above.

### Quantitative real-time PCR

Total RNA was extracted with TRIzol Reagent (Invitrogen, Carlsbad, CA, USA). Reverse transcription was performed using SuperScript™ III First-Strand Synthesis System (Invitrogen, Carlsbad, CA, USA). The primer sequences used in the experiment were listed in Table 2. Real-time PCR was performed using SYBR® Green PCR Master Mix (Roche, Mannheim, Germany) on CFX96™ Real-Time PCR Detection System (Biorad). Samples were run in triplicates. PCR conditions were 94°C for 3 min followed by 40 cycles of 94°C for 30 s, 60°C for 30 s and 72°C for 30 s, using a melting curve program and continuous fluorescence measurement. The results are reported as the relative expression after normalization of the transcript to glyceraldehyde-3-phosphate dehydrogenase (*GAPDH*; endogenous control) using the 2_T_^–ΔΔC^ method [27].

**Table 2:** Primers used in qRT-PCR

**Gene symbol**	**Primer sequence(5’-3’)**	**Product size (bp)**
Nestin (Homo Sapiens)	Forward: TGGCAAAGGAGCCTACTCCAAGAA ; Reverse: ATCGGGATTCAGCTGACTTAGCCT	111
Nestin (Rattus Norvegicus)	Forward: AGAGAAGCGCTGGAACAGAG; Reverse: AGGTGTCTGCAACCGAGAGT	234
Tuj1(Rattus Norvegicus)	Forward: AGCAGATGCTGGCCATTCAGAGTA; Reverse: TAAACTGCTCGGAGATGCGCTTGA	174
Sox2 (Rattus Norvegicus)	Forward: AAAGGAGAGAAGTTTGGAGCCCGA; Reverse: GGGCGAAGTGCAATTGGGATGAAA	113
Vimentin (Rattus Norvegicus)	Forward: AGGTGGATCAGCTCACCAATGACA ; Reverse: TCAAGGTCAAGACGTGCCAGAGAA	184
Map2 (Rattus Norvegicus)	Forward: GCAGCGCCAATGGATTTCCATACA ; Reverse: TCCGTTGATCCCGTTCTCTTTGGT	104
Gapdh(Homo Sapiens)	Forward: CATGTTCGTCATGGGTGTGAACCA; Reverse: AGTGATGGCATGGACTGTGGTCAT	160
Gapdh(Rattus Norvegicus)	Forward: AAGGGCTCATGACCACAGTC; Reverse: GTGAGCTTCCCATTCAGCTC	169

### MiRNA extraction and TaqMan assay

MiRNAs were extracted using the miRVana extraction kit (Ambion, Austin,TX, USA) according to the manufacturer’s instructions after a phenol-chloroform purification step. For quantification of *miR-125b*, 2 μg total RNA was reverse-transcribed and amplified by using the microRNA reverse transcription and detection Kit (Applied Biosystems, Inc. Foster City, CA) according to the manufacturer’s specified guidelines. All results were normalized to U6 levels that were detected by using the ABI miRNA U6 assay kit (Applied Biosystems, Inc. Foster City, CA). PCRs were normalized with the same test samples and U6 rRNA-specific primers. Significant differences in expression were determined using the Student’s *t*-test and considered significant if *P* < 0.05.

### Proliferation assay

The detection of cell proliferation has employed the incorporation of the thymidine analog BrdU (Bromodeoxyuridine, 5-bromo-2'-deoxyuridine) during DNA synthesis, followed by detection with an anti-Brdu antibody [28]. In our study, experimental groups of cells were left in the cell media pulse-labeled with 10 μM BrdU (B5002, Sigma-Aldrich, St. Louis, MO) for 16 hours and stained with an anti-BrdU antibody (B2531, Sigma-Aldrich, St. Louis, MO) [29]. For the BrdU staining, the cells were fixed with 4% paraformaldehyde in phosphate buffer at room temperature for 30 min and then washed and stored in PBS. All experiments were performed on a minimum of three litters in triplicate.

### Western blotting

NS/PCs were lysed with RIPA buffer (Sigma-Aldrich, St. Louis, MO) supplemented with proteinase inhibitor cocktail (Sigma-Aldrich, St. Louis, MO) for 30 min on ice to prepare the whole-cell lysates. The cell lysates were then clarified by centrifugation at 12,000 × *g* for 30 min. The protein concentration was measured by Bradford assay. Equivalent quantity protein lysates were electrophoresed in 10% SDS-ployacrylamide gel, and transferred to a polyvinylidene difluoride membrane (Amershame, GE, Buckinghamshire, UK). The membrane was block in tris-buffered saline (TBS) containing 10% nonfat milk and 0.05% tween-20 for 2 h at the room temperature. Primary antibodies were incubated overnight at 4°C, and primary antibodies included anti-N*estin* (SC-21248, Santa Cruz Biotechnology Inc., Santa Cruz, CA), and anti-*alpha-tubulin* (Sigma-Aldrich, St. Louis, MO). The HRP labeled secondary antibody (PIERCE, Rockford, USA) was used for 2 h at the room temperature at the dilution of 1:5000.

### Immunofluorescence

Four days after transfection, NS/PCs cells were fixed in cold 4% PFA for 30 min, followed by three washes with phosphate-buffered saline (PBS). After a 1-h blocking with 0.2% Triton X-100 and 3% goat serum in PBS, the cells were incubated with primary antibodies at 37°C for 2 hours. The primary antibodies used in this study include *Nestin* (1:400; MAB353, Millipore) , Brdu (1:500; B2531, Sigma ), *Tuj1* (1:500,05-549, Upstate), Sox2 (1:100; 481400, Life technologies ), Map-2 (1:400; M1406, Sigma), Vimentin (1:100; V6630, Sigma) overnight at 4°C . All primary antibodies are diluted in the blocking buffer (1% goat serum in PBS) after which cells are washed three times with PBS, and incubated for 1 h at room temperature with secondary antibodies. The secondary antibodies are anti-mouse IgG FITC antibody (1:200, St. Louis, MO, USA) and anti-rabbit IgG FITC antibody (1:1000, St. Louis, MO, USA) diluted in blocking buffer. Nuclei are counter-stained with Hochest 33342 (1:500; 94403, St. Louis, MO, USA). The stained cells are washed three times with PBS and visualized on Zeiss 200 inverted fluorescent microscope (Carl Zeiss, Jena, Germany). The number of immunostained cells was counted in each of three random fields per well and the fluorescence images were selected randomly. Quantification of the immunofluorescence signal was performed by measuring average intensity in individual cells according to the Image J software manual.

### Transwell migration assay

Migration assays were carried out in a 24-well transwell using polycarbonate membranes with 8-μm pores (Corning Costar, Cambridge, MA). For adherent NS/PC cells porous membrane was coated by collagen. NS/PCs cells at a density of 1 × 10^6^ cells/ml in 100 μl of medium (DMEM/F12) were placed in the upper chamber of the transwell assembly. The lower chamber contained 600 μl of medium (DMEM/F12 + 1% fetal bovine serum + 100 ng/ml SCF) [30]. After incubation at 37°C and 5% CO2 for 12 hours, the upper surface of the membrane was scraped gently to remove non-migrating cells and washed with phosphate-buffered saline. The membrane was then fixed in 4% paraformaldehyde for 15 minutes and then stained with hematoxylin and eosin. The number of migrating cells was determined by counting five random fields per well under the microscope at × 40 magnification. Assays were performed in duplicates and data was expressed as percent migration with control.

#### Statistical analysis

All values are expressed as mean ± s.d. Statistical significance (defined as P < 0.05) was evaluated with SPSS software (SPSS GmbH, Munich, Germany) using the Student's *t*-test and ANOVA .

## Abbreviations

miRNAs: MicroRNAs; mRNA: Messenger RNA; NS/PCs: Neural stem and progenitor cells; 3’-UTR: 3’ untranslated region; CNS: Central nervous system; ES cells: Embryonic stem cells; EC cells: Embryocarcinoma cells; NC: Negative control; INNC: Inhibitor negative control; BrdU: Bromodeoxyuridine; FAM: 6-carboxy-fluo-rescine; RNAi: RNA interference; SiRNA: Small interfering RNA; DMEM: Dulbecco's modified Eagle's medium; FBS: Fetal bovine serum; EGF: Epidermal growth factor; BFGF: Basic fibroblast growth factor; TBS: tris-buffered saline; PBS: Phosphate-buffered saline; FITC: Fluoresceinisothiocyanate; SCF: Stem cell factor; ANOVA: Analysis of variance.

## Competing interests

The authors declare that they have no competing interests.

## Authors' contributions

YC performed the experiments and analyzed the data. ZFX performed the statistical analysis. YNZ and JH participated in cell culture. JS, WYD and XRL helped to perform RT-PCR and the Western blot analysis. BC helped to draft the manuscript. JWD conceived of the study and participated in its design. The authors have declared that no competing interests exist. All authors read and approved the final manuscript.

## References

[B1] Gage FH Mammalian neural stem cells Science 2000 287 5457 1433 1438 10.1126/science.287.5457.1433 10688783

[B2] Lunyak VV Rosenfeld MG Epigenetic regulation of stem cell fate Hum Mol Genet 2008 17 R1 R28 36 10.1093/hmg/ddn149 18632693

[B3] Armstrong L Epigenetic control of embryonic stem cell differentiation Stem Cell Rev 2012 8 1 67 77 10.1007/s12015-011-9300-4 21808982

[B4] Spivakov M Fisher AG Epigenetic signatures of stem-cell identity Nat Rev Genet 2007 8 4 263 271 10.1038/nrg2046 17363975

[B5] Ambros V The functions of animal microRNAs Nature 2004 431 7006 350 355 10.1038/nature02871 15372042

[B6] Bartel DP MicroRNAs: genomics, biogenesis, mechanism, and function Cell 2004 116 2 281 297 10.1016/S0092-8674(04)00045-5 14744438

[B7] Li X Jin P Roles of small regulatory RNAs in determining neuronal identity Nat Rev Neurosci 2010 11 5 329 338 20354535 10.1038/nrn2739

[B8] Yi R Fuchs E MicroRNAs and their roles in mammalian stem cells J Cell Sci 2011 124 Pt 11 1775 1783 21576351 10.1242/jcs.069104 PMC3096054

[B9] Olsen PH Ambros V The lin-4 regulatory RNA controls developmental timing in Caenorhabditis elegans by blocking LIN-14 protein synthesis after the initiation of translation Dev Biol 1999 216 2 671 680 10.1006/dbio.1999.9523 10642801

[B10] Boissart C Nissan X Giraud-Triboult K Peschanski M Benchoua A miR-125 potentiates early neural specification of human embryonic stem cells Development 2012 139 7 1247 1257 10.1242/dev.073627 22357933

[B11] Le MT Xie H Zhou B Chia PH Rizk P Um M Udolph G Yang H Lim B Lodish HF MicroRNA-125b promotes neuronal differentiation in human cells by repressing multiple targets Mol Cell Biol 2009 29 19 5290 5305 10.1128/MCB.01694-08 19635812 PMC2747988

[B12] Rybak A Fuchs H Smirnova L Brandt C Pohl EE Nitsch R Wulczyn FG A feedback loop comprising lin-28 and let-7 controls pre-let-7 maturation during neural stem-cell commitment Nat Cell Biol 2008 10 8 987 993 10.1038/ncb1759 18604195

[B13] Park D Xiang AP Mao FF Zhang L Di CG Liu XM Shao Y Ma BF Lee JH Ha KS Nestin is required for the proper self-renewal of neural stem cells Stem Cells 2010 28 12 2162 2171 10.1002/stem.541 20963821

[B14] Xue XJ Yuan XB Nestin is essential for mitogen-stimulated proliferation of neural progenitor cells Mol Cell Neurosci 2010 45 1 26 36 10.1016/j.mcn.2010.05.006 20510364

[B15] Temple S Stem cell plasticity–building the brain of our dreams Nat Rev Neurosci 2001 2 7 513 520 10.1038/35081577 11433376

[B16] Goldman SA Sim F Neural progenitor cells of the adult brain Novartis Found Symp 2005 265 66 80 discussion 82-97 16050251

[B17] Kawahara H Imai T Okano H MicroRNAs in Neural Stem Cells and Neurogenesis Front Neurosci 2012 6 30 22416227 10.3389/fnins.2012.00030 PMC3298868

[B18] Lewis BP Burge CB Bartel DP Conserved seed pairing, often flanked by adenosines, indicates that thousands of human genes are microRNA targets Cell 2005 120 1 15 20 10.1016/j.cell.2004.12.035 15652477

[B19] Wei LC Shi M Cao R Chen LW Chan YS Nestin small interfering RNA (siRNA) reduces cell growth in cultured astrocytoma cells Brain Res 2008 1196 103 112 18234160 10.1016/j.brainres.2007.11.026

[B20] Jiang F Liu T He Y Yan Q Chen X Wang H Wan X MiR-125b promotes proliferation and migration of type II endometrial carcinoma cells through targeting TP53INP1 tumor suppressor in vitro and in vivo BMC Cancer 2011 11 425 10.1186/1471-2407-11-425 21970405 PMC3210504

[B21] Sahlgren CM Pallari HM He T Chou YH Goldman RD Eriksson JE A nestin scaffold links Cdk5/p35 signaling to oxidant-induced cell death EMBO J 2006 25 20 4808 4819 10.1038/sj.emboj.7601366 17036052 PMC1618100

[B22] Sahlgren CM Mikhailov A Vaittinen S Pallari HM Kalimo H Pant HC Eriksson JE Cdk5 regulates the organization of Nestin and its association with p35 Mol Cell Biol 2003 23 14 5090 5106 10.1128/MCB.23.14.5090-5106.2003 12832492 PMC162223

[B23] Jessberger S Aigner S Clemenson GD Jr Toni N Lie DC Karalay O Overall R Kempermann G Gage FH Cdk5 regulates accurate maturation of newborn granule cells in the adult hippocampus PLoS Biol 2008 6 11 e272 10.1371/journal.pbio.0060272 18998770 PMC2581629

[B24] Rakic S Yanagawa Y Obata K Faux C Parnavelas JG Nikolic M Cortical interneurons require p35/Cdk5 for their migration and laminar organization Cereb Cortex 2009 19 8 1857 1869 10.1093/cercor/bhn213 19037081 PMC2705696

[B25] Bjorklund A Lindvall O Cell replacement therapies for central nervous system disorders Nat Neurosci 2000 3 6 537 544 10.1038/75705 10816308

[B26] Johe KK Hazel TG Muller T Dugich-Djordjevic MM McKay RD Single factors direct the differentiation of stem cells from the fetal and adult central nervous system Genes Dev 1996 10 24 3129 3140 10.1101/gad.10.24.3129 8985182

[B27] Livak KJ Schmittgen TD Analysis of relative gene expression data using real-time quantitative PCR and the 2(-Delta Delta C(T)) Method Methods 2001 25 4 402 408 10.1006/meth.2001.1262 11846609

[B28] Gratzner HG Monoclonal antibody to 5-bromo- and 5-iododeoxyuridine: A new reagent for detection of DNA replication Science 1982 218 4571 474 475 10.1126/science.7123245 7123245

[B29] Cicero S Herrup K Cyclin-dependent kinase 5 is essential for neuronal cell cycle arrest and differentiation J Neurosci 2005 25 42 9658 9668 10.1523/JNEUROSCI.1773-05.2005 16237170 PMC6725732

[B30] Kimura A Ohmori T Ohkawa R Madoiwa S Mimuro J Murakami T Kobayashi E Hoshino Y Yatomi Y Sakata Y Essential roles of sphingosine 1-phosphate/S1P1 receptor axis in the migration of neural stem cells toward a site of spinal cord injury Stem Cells 2007 25 1 115 124 10.1634/stemcells.2006-0223 16990586

